# Evaluating corticosterone as a biomarker for amphibians exposed to increased salinity and ambient corticosterone

**DOI:** 10.1093/conphys/coab049

**Published:** 2021-07-03

**Authors:** Brian J Tornabene, Blake R Hossack, Erica J Crespi, Creagh W Breuner

**Affiliations:** 1 Wildlife Biology Program, W.A. Franke College of Forestry & Conservation, University of Montana, Missoula, MT 59812, USA; 2 US Geological Survey, Northern Rocky Mountain Science Center, Missoula, MT 59812, USA; 3 School of Biological Sciences, Center for Reproductive Sciences, Washington State University, Pullman, WA 99163, USA

**Keywords:** Endocrine disruption, endocrinology, frogs, salamanders, stress physiology

## Abstract

Physiological biomarkers are commonly used to assess the health of taxa exposed to natural and anthropogenic stressors. Glucocorticoid (GC) hormones are often used as indicators of physiological stress in wildlife because they affect growth, reproduction and survival. Increased salinity from human activities negatively influences amphibians and their corticosterone (CORT; the main amphibian GC) physiology; therefore, CORT could be a useful biomarker. We evaluated whether waterborne CORT could serve as a biomarker of salt stress for three free-living amphibian species that vary in their sensitivity to salinity: boreal chorus frogs (*Pseudacris maculata*), northern leopard frogs (*Rana pipiens*) and barred tiger salamanders (*Ambystoma mavortium*). Across a gradient of contamination from energy-related saline wastewaters, we tested the effects of salinity on baseline and stress-induced waterborne CORT of larvae. Stress-induced, but not baseline, CORT of leopard frogs increased with increasing salinity. Salinity was not associated with baseline or stress-induced CORT of chorus frogs or tiger salamanders. Associations between CORT and salinity were also not related to species-specific sensitivities to salinity. However, we detected background environmental CORT (ambient CORT) in all wetlands and spatial variation was high within and among wetlands. Higher ambient CORT was associated with lower waterborne CORT of larvae in wetlands. Therefore, ambient CORT likely confounded associations between waterborne CORT and salinity in our analysis and possibly influenced physiology of larvae. We hypothesize that larvae may passively take up CORT from their environment and downregulate endogenous CORT. Although effects of some hormones (e.g. oestrogen) and endocrine disruptors on aquatic organisms are well described, studies investigating the occurrence and effects of ambient CORT are limited. We provide suggestions to improve collection methods, reduce variability and avoid confounding effects of ambient CORT. By making changes to methodology, waterborne CORT could still be a promising, non-invasive conservation tool to evaluate effects of salinity on amphibians.

## Teaser

Salinization is harmful to amphibians, and waterborne corticosterone (CORT) could be a useful biomarker. Salinity was only associated with waterborne CORT for one of three amphibian species. Ambient CORT likely confounded associations and possibly influenced amphibian physiology. We provide suggestions to improve the reliability of waterborne CORT as a biomarker of salt stress.

## Introduction

It is important to evaluate tools to assess the health of wildlife species affected by anthropogenic disturbances and inform management efforts (Wikelski and Cooke, 2006). Physiological biomarkers are one such tool that is often used to gauge the responses and vulnerability of wildlife to disturbances (McCarthy and Shugart, 1990; Roy *et al.*, 1996; Wikelski and Cooke, 2006). Glucocorticoid (GC) hormones have increasingly been used as physiological biomarkers of individual and population conditions (Tarlow and Blumstein, 2007; Busch and Hayward, 2009; Hansen *et al.*, 2016). Cortisol and corticosterone (CORT) are steroid hormones secreted from the hypothalamic–pituitary–adrenal/interrenal axis (HPA/I) in vertebrates and regulate energetic balance, homeostasis and growth at baseline levels (McEwen and Wingfield, 2003). In response to acute disturbances, CORT levels often increase and redirect energy, alter behaviours and mediate life history trade-offs to increase fitness (Sapolsky *et al.*, 2000; Breuner *et al.*, 2008; Crespi *et al.*, 2013). Animals exposed to chronic disturbances typically exhibit elevated baseline CORT levels but dampened CORT responses to novel stressors, which can negatively affect fitness-related traits (Romero, 2004; Rich and Romero, 2005). Therefore, using CORT as a biomarker of physiological stress is a promising conservation tool (Cooke *et al.*, 2013; Madliger *et al.*, 2016).

Waterborne CORT is a novel, non-invasive collection method for aquatic vertebrates that provides an integrated measure reflecting CORT levels over a 1–2-h collection period (Scott and Ellis, 2007; Narayan *et al.*, 2019). Integrated measures are suggested to be more reliable indicators of stress than instantaneous measures (e.g. from plasma; Dickens and Romero, 2013). Hormones are passively diffused through permeable surfaces of an aquatic vertebrate (e.g. gills and skin) into water in a collection vessel containing the animal (Scott and Ellis, 2007; Gabor *et al.*, 2013a). For waterborne CORT to be a reliable biomarker, it should reflect endogenous CORT levels, change consistently (increase or decrease) with deteriorating environmental conditions and be robust to environmental variation (Sheriff *et al.*, 2011).

One source of environmental variation that can affect inferences from waterborne CORT is environmental CORT from organisms or anthropogenic sources (hereafter, ambient CORT; Stavreva *et al.*, 2012; Gabor *et al.*, 2018). Site waters may need to be used to collect waterborne CORT in environments with unique water quality (e.g. conductivity or pH) to avoid eliciting a stress-response by exposing native organisms to new conditions. In this situation, researchers need to measure and account for ambient CORT. To our knowledge, the pervasiveness, variability and effects of ambient CORT are not well understood (but see Stavreva *et al.*, 2012; Gabor *et al.*, 2018). Nevertheless, waterborne CORT has been used to evaluate physiological effects of contaminants, disease and invasive predators on aquatic vertebrates such as amphibians (Gabor *et al.*, 2015, 2018; Narayan *et al.*, 2019).

Waterborne CORT could also serve as a biomarker for freshwater vertebrates experiencing increased salinity from anthropogenic sources. Salinity is a persistent contaminant of freshwater ecosystems that has increased globally (Kaushal *et al.*, 2005; Dugan *et al.*, 2017). Hereafter, when using ‘salinity’, we refer explicitly to increased chloride (Cl) from sodium chloride (NaCl) salts, but we recognize that other salts (e.g. MgCl) can also negatively influence freshwater ecosystems (Harless *et al.*, 2011; Hopkins *et al.*, 2013). There are many causes of increased salinity, including application of road salts, increased irrigation for agriculture and high-salinity wastewaters from energy extraction (herafter, wastewaters; reviewed in Herbert *et al.*, 2015). Increased salinity can negatively affect the survival, growth and physiology of freshwater vertebrates such as fishes and amphibians (reviewed in Albecker and McCoy, 2017; Hintz and Relyea, 2019). Despite the negative effects of salinity, evaluations of CORT as a biomarker for free-living, freshwater organisms exposed to salinity are limited (but see Hall *et al.*, 2017, 2020).

Many amphibians are susceptible to salinity because of their porous skin and limited osmoregulatory capacity (Shoemaker and Nagy, 1977; Hintz and Relyea, 2019); however, some species are tolerant or can become adaptively tolerant to saline environments (reviewed in Hopkins and Brodie, 2015). Increased salinity from two different sources (wastewaters and road salts) has similar lethal and sublethal effects on larval amphibians in laboratory and field studies (Albecker and McCoy, 2017; Hossack *et al.*, 2018; Hintz and Relyea, 2019; Tornabene *et al.*, 2020). CORT (the predominant GC in amphibians; Macchi and Phillips, 1966) can increase with exposure to salinity (Chambers, 2011; Chambers *et al.*, 2013; Hopkins *et al.*, 2016; Hall *et al.*, 2017, 2020) and is thought to be involved in iono- and osmo-regulation of aquatic vertebrates (Warburg, 1995; McCormick, 2001; McCormick and Bradshaw, 2006). Therefore, CORT could be a useful biomarker of physiological stress and increased risk of negative health outcomes for amphibians exposed to increased salinity.

Our objective was to evaluate whether CORT could be used as a biomarker of salt stress for larval amphibians. To this end, we sought to determine the effects of increased salinity from wastewaters on baseline and stress-induced waterborne CORT of three widespread larval amphibian species that vary in their sensitivity to salinity (Hossack *et al.*, 2018, Tornabene *et al.*, 2020). All three species occur across a wide gradient of salinity from wastewaters (~0–6000 mg/l Cl dependent on species; Hossack *et al.*, 2018). We also sought to document the occurrence and variability of ambient CORT in wetlands and assessed effects on waterborne CORT of larvae. If salinity from wastewaters affects waterborne CORT, then waterborne CORT may be an effective biomarker to assess physiological stress of larval amphibians exposed to salinity. Given similarities between effects of NaCl and wastewaters on amphibians (Tornabene *et al.*, 2020), CORT could also be an effective biomarker for other sources of salt contamination.

## Materials and methods

### Ethics statement

Procedures for animal collection were approved by the University of Montana Institutional Animal Care and Use Committee (permit #024-18BHWB-050818). We collected larvae under US Fish and Wildlife special use permits #62560-16-022 and #61530-18-003; Montana Fish, Wildlife & Parks scientific collection permits #2017-104-W and #2018-083-W; and North Dakota Game and Fish collection licences #GNF04334863 and #GNF04882458.

### Study system

**Figure 1:**
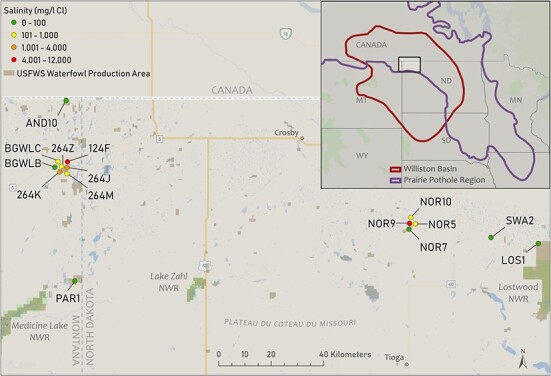
Study area including sites within US Fish and Wildlife (USFWS) Waterfowl Production Areas and National Wildlife Refuges in Montana and North Dakota, USA, where we collected waterborne, interrenal and ambient CORT samples. See Table 1 for site attributes.Baselayer sources: Esri, HERE, Garmin, SafeGraph, FAO, METI/NASA, USGS, EPA, NPS, NOAA, State of North Dakota.

We sampled waterborne CORT of larval amphibians in the Williston Basin in Montana and North Dakota, USA (Fig. 1). The study area is dominated by shortgrass prairie with limited canopy cover and few trees. The Williston Basin is one of the largest energy reserves in North America and overlaps with the Prairie Pothole Region. Legacy wastewater storage practices and recent spills have increased salinity in surface waters such as wetlands (Gleason and Tangen, 2014, Maloney *et al.*, 2017). Wastewaters and NaCl have similar effects on survival and fitness-related traits of larval amphibians despite wastewaters containing other components such as heavy metals, volatile organic compounds and radionuclides (Gleason and Tangen, 2014, Cozzarelli *et al.*, 2017).

From 2017 to 2019, we sampled from 15 fishless wetlands that spanned a gradient of salinity of 1–11 754 mg/l Cl (Table 1). We measured Cl concentrations at wetlands with Hach QuanTab Cl titration strips [ranges: 32–600 (‘low’) and 300–6000 (‘high’) mg Cl/L; accuracy ±10%; Hach Co., Loveland, CO, USA]. To measure Cl concentrations, we collected aliquots of water from around the perimeter of each wetland and mixed them prior to measurement. For any wetlands with Cl greater than 6000 mg/l, we diluted the sample by 50% with deionized water, tested Cl in the sample and multiplied the number by 2. For wetlands with Cl below detection limits of titration strips (<32 mg/l Cl), we collected 1-l water samples and Cl was measured at a US Geological Survey laboratory (Fishman and Fishman, 1989). We based site selection on previous research (Hossack *et al.*, 2017, Smalling *et al.*, 2019), occurrence of amphibians and our goal to sample across a gradient of contamination. All sampled wetlands were on US Fish and Wildlife Service lands except for two wetlands on private property (sites ‘BGWLB’ and ‘BGWLC’; Table 1).

Three species of amphibians commonly occur in our study system: barred tiger salamanders (*Ambystoma mavortium*; hereafter, tiger salamanders), northern leopard frogs (*Rana pipiens*; leopard frogs) and boreal chorus frogs (*Pseudacris maculata*; chorus frogs; Hossack *et al.*, 2018). The abundance and occurrence of amphibians in wastewater-contaminated wetlands varies with degree of contamination (Hossack *et al.*, 2018). Similarly, sensitivity to wastewaters varies among species with chorus frogs generally being the most sensitive followed by leopard frogs and then tiger salamanders (based on lethal concentration 50 tests; Tornabene *et al.*, 2020).

### Validation and collection of waterborne CORT

Waterborne CORT has been physiologically and biologically validated for many fish and amphibian species (Scott and Ellis, 2007, Narayan, 2013, Narayan *et al.*, 2019). For physiological validation, studies often compare waterborne to plasma or whole-body CORT to ensure concentrations in water correlate with endogenous concentrations of CORT (Scott and Ellis, 2007, Gabor *et al.*, 2013a, Millikin *et al.*, 2019). However, when animals are too small to obtain plasma, extracting CORT from other tissues is necessary (Millikin *et al.*, 2019, Hall *et al.*, 2020). CORT is synthesized in the interrenal gland in amphibians (Johnston *et al.*, 1967) and recovery of CORT is generally greater from interrenal glands than whole body (EJC, unpublished data). Therefore, comparing waterborne and interrenal CORT should be appropriate for physiological validation.

We collected paired baseline waterborne CORT and interrenal gland samples in 2017 to physiologically validate waterborne CORT for our study species. We tested effects of salinity on CORT of amphibians by collecting waterborne CORT samples in 2017 and 2018. In 2018, we did not sacrifice larvae to extract interrenal glands. Instead, we collected baseline and stress-induced CORT samples to assess the ability of amphibians to respond to an acute stressor (see below). We sampled 13 total sites in 2017 and 2018 (Table 1), but only three of the same sites were sampled in both years because amphibian occupancy differed and some wetlands were dry between years (Table 1). We used dipnets to collect an average of seven larvae [standard deviation (SD) = 3; range, 1–16] for each species that occurred in each wetland in each year (Table 1). We limited sampling of a species at a site to a single day within each year to avoid variation among days at a site. We collected samples from 17 June to 2 July 2017 and 12 June to 3 July 2018 when mean water temperatures were 21.8 (SD = 4.2).

To collect waterborne CORT samples (baseline and stress induced), we generally followed the methods of Gabor *et al.* (2013a). We wore nitrile gloves when handling larvae and collecting water samples and changed gloves between each larva or sample. We collected waterborne CORT samples between 1200 and 1600 h each day to avoid confounding effects of circadian rhythms on circulating CORT (e.g. Thurmond *et al.*, 1986; Wright *et al.*, 2003). We used 950-ml polypropylene containers and ~400-ml sieves made from HDPE bottles. We filled containers with 200 ml of site water from respective wetlands and placed the larva within the container, inside the sieve, immediately after capture (<3 min). Site waters were used to minimize stress responses to different water chemistries (i.e. moving larvae to water with different salinity or pH). Larvae sat in containers undisturbed for 1 h before carefully removing the larvae, mixing the water sample thoroughly and collecting 100 ml of the water for analysis. After collecting baseline samples in 2017, we measured SVL and mass and then euthanized larvae with buffered MS222 (tricaine methanesulfonate; 1 g/l). Larvae were placed in polyethylene bags. Larvae and water samples were transported to the lab in coolers with ice (held on ice for <4 h) and then held at −20°C until processing. We cleaned all containers for waterborne CORT collection between uses by rinsing them sequentially with Virkon S disinfectant (Lanxess Corporation, Pittsburgh, PA, USA), deionized water, 95% ethanol and deionized water again and then allowed them to dry.

In 2018, we used the same methods to collect baseline samples and subsequently placed larvae in 200 ml of fresh site water in clean containers to collect stress-induced waterborne CORT samples. We gently shook containers and larvae for the first 5 min to elicit an acute stress response (Glennemeier and Denver, 2002, Narayan and Hero, 2013). Larvae remained in containers for another 55 min before collecting 100 ml of the water for analysis (1 h total). Following this, we measured mass and SVL and released larvae at their point of capture. All water samples were stored at −20°C until processing.

#### Ambient CORT

To investigate and correct for ambient CORT in wetlands, we collected 100 ml of water during each visit to wetlands in 2017 and 2018 (Gabor *et al.*, 2018). Water samples for ambient CORT were collected separately from waterborne CORT samples. In 2019, to assess spatial variation in ambient CORT, we collected 100 ml of site water from five locations in each of six wetlands across a gradient of salinity (Table 1). Water samples were collected just below the water surface at evenly spaced locations along the perimeter. All water samples were stored at −20°C until processing.

To evaluate their influence on ambient CORT, we measured several biotic and abiotic variables of wetlands. We measured Cl as described above and specific conductance with metres (Hanna Instruments HI98130). Wetland area (based on length and width measurements) and percent shallows (≤0.50-m depth) were estimated at each site. Using a regression of Cl and amphibian abundance from the same study area (from 2015 to 2017), we estimated amphibian abundance across all species for our wetlands (Hossack *et al.*, 2018). We also evaluated whether ambient CORT was associated with mean waterborne CORT (baseline or stress induced) pooled across the three species we studied.

**Table 1:** Locations and attributes of wetlands, ordered from highest to lowest salinity (mg/l Cl), where we collected waterborne CORT, larvae to extract interrenal CORT, water samples to extract ambient CORT or a combination of the aforementioned

			Larvae									
			2017			2018				Salinity (mg/l Cl)		
Site	State	Location	T	L	C	T	L	C	Water	2017	2018	2019
LOS1	ND	Lostwood NWR					11	9			1.46	
SWA2	ND	Swanson WPA		9	9					5.06		
BGWLB	MT	Private	3			9			y	6.81	18.94	<32
AND10	MT	Anderson WPA						9			9.72	
PAR1	MT	Pary WPA		7		6	10		y	30	101	41
NOR7	ND	Norman WPA	8			1	16			44.33	41	
NOR5	ND	Norman WPA						9			101	
BGWLC	MT	Private	8								445	
264M	MT	Rabenberg WPA							y			445
264Z	MT	Rabenberg WPA				8					552	
NOR10	ND	Norman WPA		5	9					727		
264K	MT	Rabenberg WPA				10			y	1001.50		813
264J	MT	Rabenberg WPA	3						y	2494.50		2225.00
NOR9	ND	Norman WPA	9	4						4103.00		
124F	MT	Rabenberg WPA							y			11 754.00

For ‘State’: ND indicates North Dakota; MT, Montana.

For ‘Location’: WPA indicates US Fish and Wildlife Service Waterfowl Production Area; NWR, National Wildlife Refuge.

For ‘Larvae’: T = barred tiger salamanders, L = northern leopard frogs, C = boreal chorus frogs; paired baseline and interrenal CORT samples were collected in 2017 and paired baseline and stress-induced CORT samples were collected in 2018.

For ‘Water’: y = replicate water samples collected on the same day in 2019 from several wetlands to assess variation in ambient CORT within wetlands.

Blank cells indicate that no samples were taken at that site in that year.

### CORT extraction and quantification

#### Interrenal tissue extraction

We followed the methods of Hall *et al.* (2020) to extract CORT from interrenal tissue. Briefly, we thawed frozen larva and dissected the interrenal gland. We weighed interrenal tissue, placed it in a borosilicate vial with 0.5 ml of ice-cold phosphate-buffered saline and homogenized with a dispersing instrument (Ultra-Turrax T10; VWR, Radnor, Pennsylvania, USA). We used ether-lipid extractions to purify CORT. We used trial samples to optimize resuspension volumes prior to resuspending samples for this study. Samples were resuspended in 95% enzyme immunoassay (EIA) buffer/5% ethanol and volumes were based on mass of interrenal glands: 200 μl for <25 mg, 300 μl for 25–50 mg, 600 μl for 50–75 mg, 900 μl for 75–100 mg and 1200 μl for >100 mg. The mean mass of interrenal glands was 39.2 mg (SD = 35.6).

#### Waterborne CORT extraction

We used solid-phase extraction to concentrate CORT from water samples (adapted from Earley and Hsu, 2008, Gabor *et al.*, 2013a). First, we filtered water samples though quantitative grade filter paper (coarse Q8 filter paper; Fisher Scientific, Hampton, NH, USA) to remove large organic matter. We concentrated hormones using C18 solid phase extraction columns (Sep-Pak Vac C-18, 500 mg, 3.0 ml; Waters Corporation, Milford, MA, USA) and a 12-port solid phase extraction manifold (Vac Elut 12; Agilent Technologies, Santa Clara, CA, USA). Columns were primed with 4 ml of HPLC-grade methanol followed by 4 ml of double-distilled water. We transferred samples from storage bottles through columns using Tygon tubing (Saint-Gobain formulation 2475; Saint-Gobain Performance Plastics, Courbevoie, France) and rate of flow was held at ~2 ml/min. We used 4-ml distilled water to purge salts from columns and then eluted hormones into borosilicate vials with 4-ml methanol. We evaporated methanol from eluted hormone samples by placing them in a 37°C water bath and using an evaporating manifold with a gentle stream of nitrogen.

We optimized reconstitution volumes with trial samples, prior to processing field samples, because larval body sizes were larger in the present study. Samples were resuspended in 95% EIA buffer/5% ethanol and volumes were based on mass of larvae: 4 ml for <4 g, 8 ml for 4–10 g and 10 ml for >10 g. We used similar methods to quantify ambient CORT from water samples. Following optimization of trial samples, we reconstituted ambient CORT samples with 1 ml of 95% EIA buffer/5% ethanol. Any samples outside the assay standard curve (if ≤20% bound) were diluted (typically 1:2–1:4) and rerun. Resuspended samples were stored at −20°C until processing.

#### CORT quantification

We used EIAs to quantify the amount of CORT in interrenal, ambient and waterborne samples (Cayman Chemicals Inc., Ann Arbor, MI, USA). We followed manufacturer procedures, including reading each plate at 414 nm (5–7-nm bandwidth; Multiskan Ascent, Thermo Fisher Scientific; Waltham, MA, USA), and ran samples in duplicate. To analytically validate our assays, we assessed parallelism of a serial dilution to the standard curve, determined quantitative recovery and assessed intra- and inter-assay variations. We pooled 150 μl of each interrenal sample or 100 μl of each baseline waterborne hormone sample separately per species to assess parallelism and recovery. We assessed parallelism with a linear regression of percent CORT bound against pg/ml concentrations for eight serial dilutions of the standard curve (~1:1–1:610) and five serial dilutions of each pooled sample (1:1–1:16). Dilution curves (waterborne and interrenal gland samples for each species) were parallel to the standard curve (comparison of slopes: *F* < 2.94 and *P* > 0.120). For quantitative recovery, we ran unmanipulated pooled samples (for each species and type) and spiked five aliquots of each pooled sample with one of five EIA standards. We were unable to assess quantitative recovery for chorus frog interrenal samples because of limited pooled-sample volume. We used linear regression to assess linearity of observed and expected values. Expected and observed values were linear for each comparison (*P* < 0.001 and R^2^ > 0.984; Supplementary Table 1). Recovery was calculated by dividing observed by expected concentrations (Millikin *et al.*, 2019) and minimum recovery ranged from 79–128%. Sensitivity of plates ranged from 8.81–42.95 pg/ml and all samples had higher concentrations than sensitivity of respective plates. We included positive controls in each of 16 EIA plates to assess inter- and intra-assay variations. Mean intra-assay variation was 9.52% (SD = 3.60%) and inter-assay variation was 15.48%.

After multiplying waterborne CORT values by resuspension volumes, we multiplied values by two because we only analysed half of the water sample larvae sat in during collection (100 of 200 ml). We subtracted ambient CORT from larval waterborne CORT values before dividing by holding time (typically 1 h) to yield waterborne CORT release rates in pg/h. Studies often account for size by dividing waterborne CORT release rates by mass or snout-vent length (e.g. pg/SVL/h), but this can elicit artificial differences among groups. Dividing by size can inflate or deflate CORT per unit mass because of the allometric relationship between gill and integument surface area and mass (Scott and Ellis, 2007, Scott *et al.*, 2008, Archard *et al.*, 2012). Therefore, we included ln-transformed mass as a covariate in models to account for the influence of mass on ln-transformed waterborne CORT (Archard *et al.*, 2012). For interrenal gland samples, we multiplied CORT concentrations from EIAs (pg/ml) by resuspension volumes and divided by mass of the interrenal gland to obtain pg/mg CORT.

Ambient CORT was greater than baseline or stress-induced CORT for 17% of samples, which resulted in negative CORT values (herein, negative release rates) similar to a previous study (Gabor *et al.*, 2018). We observed negative release rates at most sites (80%), but the majority occurred at three sites (sites ‘BGWLB’, ‘BGWLC’ and ‘AND10’; Fig. 1). The most negative release rates were from salamanders (21% of observations for baseline and 31% for stress induced) followed by chorus frogs (20% and 18%, respectively) and leopard frogs (10% and 0%, respectively). We added a constant to all CORT values to make release rates positive prior to ln-transformation (see below under Statistical analyses). Trends in our study were qualitatively similar whether negative release rates (after adding a constant to all values) were included or not (see Supporting Materials: Tables for Models Omitting Negative Release Rate Observations). We chose to retain these observations, contrary to a previous study that excluded them (Gabor *et al.*, 2018), to include the natural variation we observed.

### Statistical analyses

#### Ambient CORT

To understand factors influencing variation in ambient CORT among sites, we separately tested the influence of nine abiotic and biotic variables on ambient CORT in wetlands from 2017 to 2019 (Supplementary Table 2). Variables included mean baseline and stress-induced CORT of larvae, salinity, estimated amphibian density across all species, area, percent shallows, site identity, specific conductivity and year. We used univariate generalized linear mixed models (GLMMs) in the package ‘nlme’ to test the influence of abiotic or biotic variables on ambient CORT (Pinheiro *et al.*, 2017). We included site as a random effect to account for several measurements of CORT from the same site within and among years. Although we measured spatial variation in ambient CORT within wetlands in 2019, we only included mean ambient CORT values from each site for the among site analysis. To understand the factors influencing variation within sites in 2019, we used univariate linear models to test the effects of six abiotic and biotic variables on coefficient of variance of ambient CORT in wetlands (Supplementary Table 2). Variables included salinity, area, percent shallows, max depth, perimeter and specific conductivity.

To test whether the probability of a larva having a measured negative release rate was related to ambient CORT in wetlands, we used binomial GLMMs with a logit link (package ‘lme4’; Bates *et al.*, 2014). We used a Bayesian generalized linear model (package ‘arm’) for chorus frogs because of separation in the data and issues with model convergence when using GLMMs (Gelman *et al.*, 2008, Gelman *et al.*, 2020). We tested each species and measure of CORT separately (baseline or stress induced), which resulted in six total comparisons. We included a random effect of larva nested within site to account for several samples being taken from each site.

#### Waterborne and interrenal CORT

Using simple linear regression, we physiologically validated our method and tested the relationship between baseline waterborne and interrenal CORT for each species. We included ln-transformed mass as a covariate to account for the influence of mass on CORT. We ln-transformed interrenal and waterborne CORT.

To determine variation in baseline and stress-induced CORT, we calculated the coefficients of variance within and among wetlands, per species. We used a paired *t*-test to compare baseline with stress-induced samples from 2018 and ensure there were changes in CORT following an acute challenge (gentle shaking). We used analysis of variance to test for differences in baseline and stress-induced CORT among species.

To test the influence of salinity on waterborne CORT, we used maximum-likelihood methods to fit GLMMs (package ‘nlme’) and used multimodel inference (package ‘MuMIn’; Bartón, 2010, Pinheiro *et al.*, 2017) separately for each species and measure of CORT (baseline or stress induced). Baseline CORT included observations from 2017 and 2018. Stress-induced CORT included only observations from 2018, when collections began. We included a random effect of larvae nested within sites to account for multiple samples being taken from each wetland. Within our models, we weighted baseline and stress-induced CORT rates by the inverse of their coefficient of variance (between duplicate EIA samples) calculated during quantification. Therefore, observations with high variance among samples in EIAs would be less heavily weighted in the model and vice versa.

For multimodel inference, we first created global models for each species × CORT comparison (six global models) that included salinity, ambient CORT, mass and a mass × salinity interaction as predictors. We included ln-transformed mass and a mass × salinity interaction because of interactive effects of salinity and mass on waterborne CORT of larval leopard frogs in a previous study (BJT, unpublished data). To improve numerical stability and reduce multicollinearity, we centred and scaled all predictor variables by subtracting the mean and dividing by the SD.

We separately created sets of all possible sub-models and determined the top models for each species × CORT combination. Importantly, all model combinations were ecologically relevant. We used Akaike’s Information Criterion for small sample sizes to compare models (AICc; Burnham and Anderson, 2004, Mazerolle, 2016). For model averaging, we included all models with AIC weight (AIC_W_) > 0.001 and weighted parameter estimates according to AIC_W_. We assessed multicollinearity in top models for each species × CORT combination by calculating variance inflation factors (VIFs) and did not detect strong multicollinearity in any top models (VIF < 3.09; Fox *et al.*, 2012). All aforementioned analyses were conducted in program R (v4.0.3; R Core Team, 2019).

## Results

### Ambient CORT

**Figure 2:**
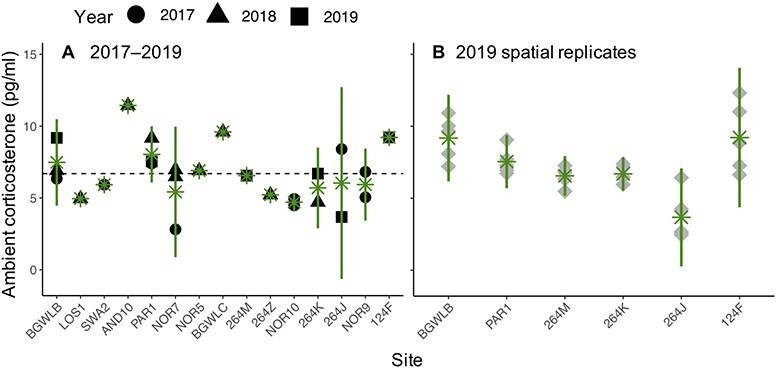
(A) Ambient CORT (pg/ml) detected in wetlands in 2017–2019. Green asterisks indicate means (+/−95% confidence intervals) for wetlands sampled in multiple years (e.g. BGWLB), or multiple times within years (e.g. NOR9), or both (e.g. NOR7). For 2019, when we collected spatial replicates within each wetland to assess variance, only mean values are presented. The dotted line indicates the grand mean among all wetlands and years. (B) Ambient CORT detected in six wetlands sampled at five points evenly around the wetland perimeter in 2019. Green asterisks indicate means (+/− 95% confidence intervals) within each wetland.

Ambient CORT was detected in all wetlands; however, it varied widely among wetlands and years and within wetlands in 2019 (Fig. 2A and B). None of the abiotic or biotic variables we measured were associated with mean or variance in ambient CORT (among and within wetlands, respectively; Supplementary Table 2). Mean ambient CORT in wetlands among years was 6.69 pg/ml (SD = 2.03 pg/ml). Among wetlands and years, coefficient of variance for ambient CORT was 30.29%. For wetlands sampled from six locations in 2019, within-wetland coefficient of variance was 20.96% (SD = 14.38%) and ranged from 8.67–46.32%.

**Figure 3:**
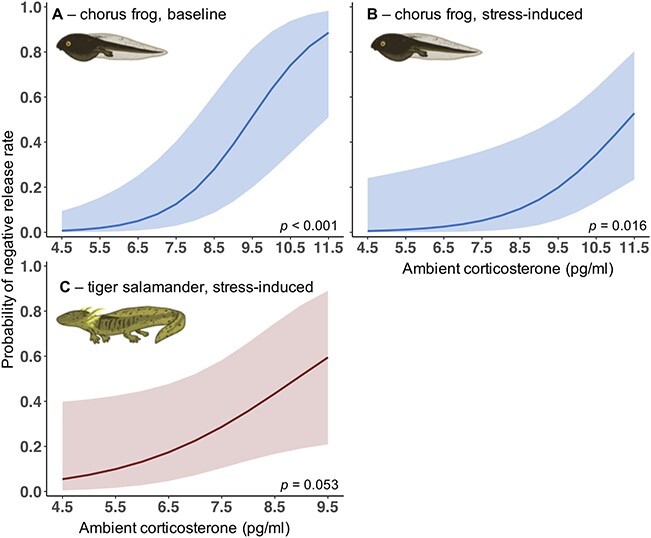
Probability of detecting a negative waterborne CORT release rate (pg/h) increased with increasing ambient CORT (pg/ml) for boreal chorus frogs baseline (A) and stress-induced (B) CORT and barred tiger salamanders stress-induced (C) CORT. Only relationships that were statistically significant are shown (Table S3).

Increasing ambient CORT increased the probability of a measured negative release rate, but associations varied among species and between baseline and stress-induced CORT (Fig. 3 and statistics in Supplementary Table 3). The probability of a negative release rate increased with ambient CORT for baseline and stress-induced CORT of chorus frogs and marginally for stress-induced CORT of tiger salamanders (Fig. 3A–C). However, ambient CORT was not associated with probability of a negative release rate for baseline CORT of tiger salamanders or leopard frogs (Supplementary Table 3). No negative release rates occurred for stress-induced CORT of leopard frogs.

### Waterborne and interrenal CORT

**Figure 4:**
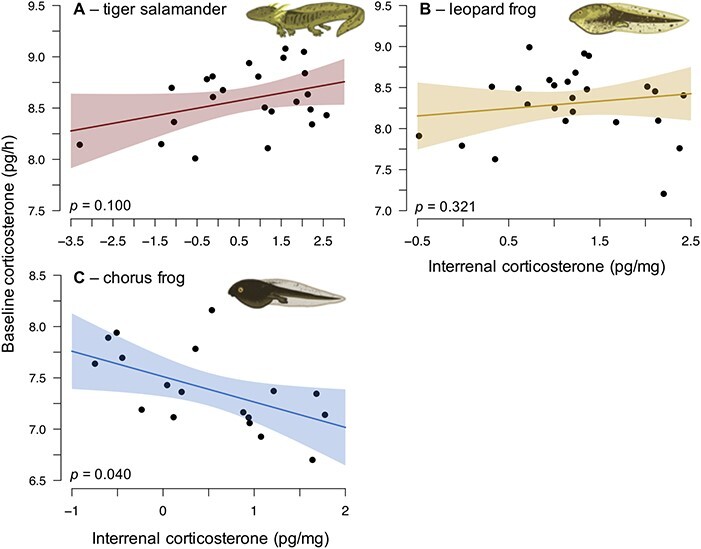
Waterborne baseline CORT (pg/h; ln transformed) was not associated with interrenal CORT (pg/mg; ln transformed) for barred tiger salamanders (A) or northern leopard frogs (B) but baseline CORT was negatively correlated with interrenal CORT for boreal chorus frogs (C).

The only association between interrenal and baseline waterborne CORT was a negative relationship for chorus frogs (Fig. 4A–C; Table 2). Mean stress-induced CORT was higher than mean baseline CORT for leopard (*t_36_* = −2.54, *P* = 0.015) and chorus frogs (*t*_26_ = −4.39, *P* < 0.001) but not for tiger salamanders (*t*_41_ = −0.88, *P* = 0.380; Supplementary Table 4). Within and among sites, coefficients of variance for baseline and stress-induced waterborne CORT were generally low (<9.62%; Table 3). Leopard frogs had the highest coefficients of variance within and among sites followed by chorus frogs and tiger salamanders (Table 3). Leopard frogs had the highest baseline (*F*_2,169_ = 57.71, *P* < 0.001) and stress-induced waterborne CORT (*F*_2,103_ = 37.07, *P* < 0.001) followed by tiger salamanders and then chorus frogs.

**Table 2:** Summary statistics (*β* and SE in natural-log scale) for linear models assessing the relationship between waterborne baseline CORT (pg/h) and interrenal CORT (pg/mg) for barred tiger salamanders (*n* = 23), northern leopard frogs (*n* = 25) and boreal chorus frogs (*n* = 18)

Species	Variable	*β*	SE	*t*	*p*	Adj. *R*^2^
Tiger salamander	Intercept	8.15	0.16	50.59	<0.001	0.289
	Interrenal CORT	0.07	0.04	1.73	0.100	
	Mass	0.20	0.08	2.53	0.020	
Leopard frog	Intercept	7.62	0.26	29.55	<0.001	0.268
	Interrenal CORT	0.11	0.11	1.02	0.321	
	Mass	0.32	0.10	3.28	0.003	
Chorus frog	Intercept	7.69	0.15	52.06	< 0.001	0.361
	Interrenal CORT	−0.23	0.10	−2.25	0.040	
	Mass	0.24	0.18	1.34	0.202	

Mass was included as a covariate to account for differences in baseline CORT release rates based on size. Interrenal and baseline CORT and mass were natural-log-transformed.

SE indicates standard error.

**Table 3:** Mean SD within-site coefficient of variance (%) and among site coefficient of variance (%) for baseline and stress-induced waterborne CORT of barred tiger salamander, northern leopard frog and boreal chorus frog larvae

	Baseline		Stress induced	
	Within	Among	Within	Among
Tiger salamander	3.19 (1.51)	5.49	2.84 (0.89)	4.89
Leopard frog	6.40 (3.20)	9.47	6.89 (3.38)	9.62
Chorus frog	4.03 (1.24)	7.02	4.11 (0.78)	6.31

Among site, coefficient of variance does not have SD because it is a single value calculated from baseline or stress-induced CORT measures.

**Figure 5:**
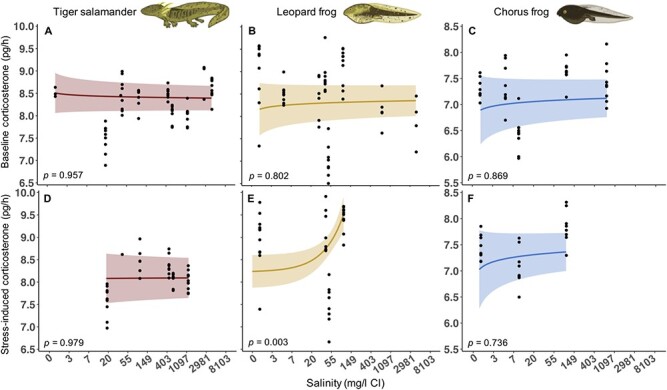
Mean (±95% confidence intervals) baseline (A–C) and stress-induced waterborne CORT (pg/h; D–F) of larval barred tiger salamanders (A and D), northern leopard frogs (B and E) and boreal chorus frogs (C and F) exposed to a gradient of salinity (mg/l Cl). Only mean stress-induced CORT of leopard frogs increased with increasing salinity. Baseline and stress-induced CORT and salinity are ln transformed, but the *x*-axis was back transformed for interpretation. Note that baseline and stress-induced CORT of chorus frogs (C and F) are on a different scale than those of tiger salamanders and leopard frogs.

Salinity was positively associated with stress-induced waterborne CORT of leopard frogs but not any other waterborne CORT measure across the three species (Fig. 5A–F and Supplementary Tables 5–9). Although included in some top models, ambient CORT and the mass × salinity interaction were not associated with baseline or stress-induced waterborne CORT of any species (Supplementary Tables 5–9). Mass was included in all top models (Supplementary Tables 5–7) and was strongly, positively correlated with baseline and stress-induced waterborne CORT for all species, except for stress-induced waterborne CORT of chorus frogs (Supplementary Tables 8 and 9).

## Discussion

We evaluated whether waterborne CORT could be used as a biomarker of physiological stress for larval amphibians exposed to increased salinity. Our model study system included three species with variable sensitivity occurring across a wide gradient of salinity. A useful biomarker would show a reliable signal with increasing salinity, represent endogenous CORT of animals and be robust to environmental variation. Of the three species in our study, only stress-induced waterborne CORT of leopard frogs increased with salinity. Associations were not related to species-specific sensitivities of amphibians to salinity. Interrenal and waterborne CORT were only correlated for one of three species. However, ambient CORT was prevalent, highly variable and potentially obscured our ability to detect trends. Ambient CORT may have also influenced CORT physiology of larvae. Using the current methods, waterborne CORT may not be a reliable biomarker of physiological stress for amphibians exposed to salinity. We provide suggestions to improve methodology, evaluate confounding effects, minimize variation and improve the reliability of CORT as a biomarker for amphibians exposed to salinity.

There are several reasons we may not have detected an effect of salinity on waterborne CORT compared with previous studies that found effects of salinity on whole-body, interrenal and plasma CORT of amphibians (Chambers, 2011, Chambers *et al.*, 2013, Hopkins *et al.*, 2016, Hall *et al.*, 2020). First, as is common in CORT physiology, relationships between CORT and salinity may be affected by environmental conditions, species identity and life history stage (Breuner *et al.*, 2008, Crespi *et al.*, 2013). Some studies have found that salinity increases CORT of amphibians, but other studies have found decreased CORT or no relationship (Burraco and Gomez-Mestre, 2016, Hopkins *et al.*, 2016, Hall *et al.*, 2017). Inconsistencies among studies could result from salinity dysregulating the HPI axis of amphibians as they grow and develop. Salinity can reduce normal increases in CORT with increasing growth and development dependent on concentration (Denver *et al.*, 2002; BJT unpublished data). Second, adaptive tolerance and habituation to salinity stress can also influence CORT responses of amphibians (Cyr and Romero, 2009; Hopkins *et al.*, 2016). Over the past 50 years, some populations in our study area have likely been exposed to salinity from wastewaters (Gleason and Tangen, 2014) and adaptive tolerance to salinity can occur within 40–70 years (Brady, 2012). Therefore, tolerance and physiological adaptation could have influenced our findings. Third, our sample sizes may have limited our ability to detect relationships between salinity and waterborne CORT if they existed. Sample sizes larger than those in our study may be necessary because of high variability documented in ambient CORT and inherent to waterborne CORT data (Gabor *et al.*, 2016). Fourth, ambient CORT led to negative release rates for chorus frogs and tiger salamanders, which had the lowest waterborne CORT release rates. We only detected an association between salinity and waterborne CORT for leopard frogs. This association may have been detectable because the species had the highest waterborne CORT, which may have limited confounding effects of ambient CORT.

Ambient CORT could have confounded associations between waterborne CORT and salinity. High ambient CORT leading to negative release rates also occurred in a study of amphibians exposed to effluents (Gabor *et al.*, 2018). Negative release rates could result from a suppression of endogenous CORT production due to high ambient CORT. For example, equilibrium effects could occur wherein larvae passively take up ambient CORT and downregulate CORT synthesis, circulation and diffusion. Indeed, several species of adult and larval amphibians can take up CORT through their skin and gills (e.g. Wack *et al.*, 2010; Middlemis Maher *et al.*, 2013; Gabor *et al.*, 2019). Treatment with synthetic CORT similarly inhibits endogenous CORT production and suppresses HPA/I activity (Andrews *et al.*, 2012, Paragliola *et al.*, 2017). Equilibrium effects, physiological effects of ambient CORT and variation in ambient CORT within and among wetlands may have therefore influenced our ability to detect associations between salinity and CORT if they existed.

We were unable to identify the source of ambient CORT in our system because none of the environmental variables we measured were related to ambient CORT. Likewise, and opposed to a previous study (Gabor *et al.*, 2018), we did not find an association between mean baseline CORT of amphibians and ambient CORT. One wetland (site ‘124F’) has such high salinity from historical wastewater contamination (~12 000 mg/l Cl in 1989 and 2019; Gleason and Tangen, 2014) that it likely has not hosted amphibians or fish for decades—yet, it still had high ambient CORT relative to other wetlands. Ambient CORT could have originated from other wildlife such as birds or ungulates (Gabor *et al.*, 2018). Our study sites in the Prairie Pothole Region have abundant waterfowl and shorebirds that frequent contaminated and uncontaminated wetlands. Future studies could use gas chromatography–mass spectrometry (GC–MS) to determine if ambient CORT was cortisol, CORT or another interfering (cross-reactive) compound. Although we did not have the resources to do GC–MS, strong parallelism in our analytical validation suggests it was not a matrix effect (i.e. contamination altering chemistry of EIAs). The occurrence, sources and variability in ambient CORT should be evaluated in subsequent studies given the effects and prevalence of ambient CORT in our study.

Our findings suggest that ambient CORT may accumulate, be more persistent in the environment than previously thought and influence CORT physiology of free-living amphibians. Other steroid hormones such as oestrogen and testosterone also occur in surface waters and can be persistent (Deksissa, 2008, Boulanger, 2012). While human activities (e.g. effluents) release hormones into surface waters, natural secretions from aquatic vertebrates seemingly also contribute (Gabor *et al.*, 2018). Testosterone and oestrogen in surface waters is well reported because of their negative effects on reproduction, and feminization, of amphibians and fishes (e.g. Sumpter and Johnson, 2005, Säfholm *et al.*, 2014). To our knowledge, CORT is not often reported (but see Stavreva *et al.*, 2012; Gabor *et al.*, 2018). Nevertheless, ambient CORT confounded our ability to detect trends and increased negative release rates in our study.

In our system, amphibians and other aquatic vertebrates are passively exposed to ambient CORT in the environment. Although effects of ambient CORT on free-living amphibians are largely unknown, exposure to ambient CORT might lead to downregulation of CORT synthesis and dysregulation of the HPI axis and might have negative effects on fitness-related traits (e.g. growth and survival). Exogenous CORT applied in housing waters affects growth, development and behaviour of early stage larvae and accelerates metamorphosis of late stage larvae (reviewed in Crespi and Denver, 2004; Belden *et al.*, 2005). The effects of ambient CORT are likely context specific and dependent upon temporal and spatial variation, duration and intensity of exposure and developmental stage of larvae. For example, persistent exposure might lead to chronic negative effects or habituation (Cyr and Romero, 2009) while transient exposure could have limited effects. Alternatively, effects of ambient CORT could be minimal because amphibians have likely been exposed to various concentrations of ambient CORT throughout their evolution. Further research is necessary to determine the effects of ambient CORT on physiology and fitness-related traits of free-living amphibians.

Interrenal and waterborne CORT were only correlated with each other for chorus frogs. Two factors may explain the lack of association in the other two species. First, waterborne and interrenal CORT may be innately different and not strongly correlated. Waterborne CORT is an integrated measure (1–2 h of collection). Interrenal CORT may be an instantaneous measure because CORT is a steroid hormone that is released immediately following synthesis and cannot be stored (Hadley and Levine, 2007). The two measures of CORT may therefore also have different biological meanings and require different interpretations and may not be appropriate to validate against one another (Millikin *et al.*, 2019). Despite weak or no correlation between interrenal and waterborne CORT in this study, previous studies have illustrated that waterborne CORT is biologically meaningful for many species and correlated with plasma or whole-body CORT levels (reviewed in Baugh *et al.*, 2018; Narayan *et al.*, 2019). Second, equilibrium effects occurring from high ambient CORT could have confounded relationships between interrenal and waterborne CORT. Taking up ambient CORT could decrease production in the interrenal gland, circulation in blood and secretion of CORT into water; however, the timing of changes among these processes is unclear. Higher waterborne CORT was associated with lower interrenal CORT for chorus frogs in our study, which could be an artefact of the effect of ambient CORT.

There are several modifications to methods that could reduce variation and confounding effects of ambient CORT. First, given the inherent variation in waterborne CORT and confounding effects of ambient CORT, future studies should strive to collect a higher number of samples per site than in our study (Gabor *et al.*, 2016). A charcoal filter could be used to strip ambient hormones from site waters for CORT collection. Although this might decrease negative release rates in calculations, it would not eliminate effects of ambient CORT on amphibian CORT physiology that occurred beforehand. Mixing waters collected from around each wetland, to use for waterborne CORT collection of all larvae in that wetland, could also reduce variability in ambient CORT. We were unable to use spring water, which is common in waterborne CORT studies (e.g. Gabor *et al.*, 2013a,b, 2015), because spring water could have exposed larvae in our study to novel water quality conditions and elevated CORT levels. Using spring water and matching relevant water quality conditions (e.g. salinity and pH) could be a useful alternative but would be time-consuming and could add other sources of variation. Other physiological biomarkers could be paired with waterborne CORT, serve as downstream metrics of chronic stress and may assist gauging the influence of salinity on amphibians. Other markers associated with CORT include changes in water retention and body index (e.g. Hall *et al.*, 2017), microbiota (Goff *et al.*, 2020), glucose, haematocrit, immune response or telomere length (Breuner *et al.*, 2013).

## Conclusions

Salinity is a pervasive anthropogenic disturbance that has negative effects on aquatic species. It is therefore imperative to identify biomarkers, ensure their reliability and evaluate the health of aquatic vertebrates exposed to salinity. Ambient CORT was prevalent and seemingly confounded associations in our analysis and influenced CORT physiology of larvae. Further investigations are warranted to document the occurrence and sources of ambient CORT in waterbodies, as well as to understand the effects of ambient CORT on aquatic vertebrates. Future studies could improve the reliability of waterborne CORT by making modifications to methodology, evaluating confounding effects and minimizing sources of environmental variation as we have described. By making changes to methodology, waterborne CORT could still be a promising, non-invasive conservation tool to evaluate effects of salinity and other disturbances, on larval amphibians.

## Funding

This work was supported by the University of Montana, the Nelson Schwab Family Foundation, the US Geological Survey (USGS RWO #103 to B.R.H.), Prairie Biotic Research, the Montana Chapter of The Wildlife Society and the National Science Foundation (NSF-DEB 1754474 to E.J.C.).

## Author contributions

B.J.T., B.R.H. and C.W.B. conceived the study. B.J.T., B.R.H., C.W.B. and E.J.C. designed the study. B.J.T. collected the data and conducted the statistical analyses. B.J.T. wrote the manuscript with revisions and input from B.R.H., E.J.C. and C.W.B. B.J.T., B.R.H., C.W.B. and E.J.C. contributed to interpretation of the data and gave final approval.

## Supplementary Material

2TornabeneFieldCORTSupportingMaterialsR_Revision_coab049
