# Supplementary material for: Evaluating corticosterone as a biomarker for amphibians exposed to increased salinity and ambient corticosterone
Source: Conserv Physiol. 2021 Jul 3;9(1):coab049. doi: 10.1093/conphys/coab049 (PMC8254138; doi:10.1093/conphys/coab049)
Supplement: 2TornabeneFieldCORTSupportingMaterialsR_Revision_coab049 [file 2tornabenefieldcortsupportingmaterialsr_revision_coab049.docx]

**Supplementary Materials for:**

**Evaluating Corticosterone as a Biomarker for Amphibians**

**Exposed to Increased Salinity and Ambient Corticosterone**

Brian J. Tornabene, Blake R. Hossack, Erica J. Crespi, and Creagh W. Breuner

**TABLES**

**Supplementary Table 1.** Summary statistics describing quantitative recovery of waterborne (“Water”) and interrenal gland (“Interrenal”) corticosterone samples for each species.

| **Species** | **Type** | ***df*** | ***F*** | ***p*** | ***R*^2^** | **Recovery** |
| --- | --- | --- | --- | --- | --- | --- |
| Tiger salamander | Water | 1,3 | 630.9 | < 0.001 | 0.994 | 99.2 |
|  | Interrenal | 1,3 | 604.1 | < 0.001 | 0.993 | 79.4 |
| Leopard frog | Water | 1,3 | 241.1 | < 0.001 | 0.984 | 101.4 |
|  | Interrenal | 1,3 | 522.5 | < 0.001 | 0.992 | 87.9 |
| Chorus frog | Water | 1,3 | 613.6 | < 0.001 | 0.993 | 127.7 |
|  | Interrenal | ND | ND | ND | ND | ND |

Quantitative recovery is calculated as observed divided by expected values and minimum recovery for each comparison is presented as a percent (%).

Quantitative recovery of interrenal corticosterone samples of chorus frogs was not determined (ND) because of limited sample volumes.

**Supplementary Table 2.** Summary statistics for *F*-tests of univariate linear mixed models investigating the influence of abiotic and biotic variables on ambient corticosterone (“Ambient”) or coefficient of variance of ambient corticosterone (“Variance”) detected in wetlands in Montana and North Dakota, USA.

| **Type** | **Variable** | ***df*** | ***F*** | ***p*** |
| --- | --- | --- | --- | --- |
| Ambient | Average baseline corticosterone | 1,17 | 0.005 | 0.946 |
|  | Average stress-induced corticosterone | 1,8 | 0.007 | 0.935 |
|  | Salinity | 1,23 | 0.529 | 0.486 |
|  | Estimated amphibian density | 1,23 | 0.318 | 0.586 |
|  | Estimated area | 1,23 | 0.147 | 0.710 |
|  | Percent shallows | 1,23 | 0.023 | 0.883 |
|  | Site | 14,10 | 1.521 | 0.255 |
|  | Specific conductivity | 1,23 | 0.799 | 0.395 |
|  | Year | 2,22 | 1.415 | 0.298 |
| Variance | Salinity | 1,4 | 0.590 | 0.485 |
|  | Percent shallows | 1,4 | 1.505 | 0.287 |
|  | Estimated area | 1,4 | 2.161 | 0.216 |
|  | Max depth | 1,4 | 1.138 | 0.346 |
|  | Perimeter | 1,4 | 2.789 | 0.237 |
|  | Specific conductivity | 1,4 | 0.498 | 0.519 |

“Site”, “Year”, and “Wetland size” are categorical variables and all others are continuous.

**Supplementary Table 3.** Summary statistics (*β* and standard error [SE] in logit scale) from generalized linear mixed models investigating relationships between probability of detecting a negative waterborne corticosterone release rate (pg/h; baseline or stress-induced) and ambient corticosterone (pg/ml) for each amphibian species.

| **Species** | **Measure** | **Variable** | ***β*** | **SE** | ***z*** | ***p*** |
| --- | --- | --- | --- | --- | --- | --- |
| Tiger salamander | Baseline | Intercept | -4.216 | 2.811 | -1.50 | 0.134 |
|  |  | Ambient CORT | 0.001 | 0.002 | 0.76 | 0.450 |
|  | Stress-induced | Intercept | -5.766 | 2.653 | -2.17 | 0.030 |
|  |  | Ambient CORT | 0.003 | 0.002 | 1.93 | 0.053 |
| Leopard frog | Baseline | Intercept | -0.342 | 2.415 | -0.14 | 0.887 |
|  |  | Ambient CORT | -0.002 | 0.002 | -0.77 | 0.441 |
|  | Stress-induced | Intercept | ND | ND | ND | ND |
|  |  | Ambient CORT | ND | ND | ND | ND |
| Chorus frog | Baseline | Intercept | -9.425 | 2.546 | -3.70 | < 0.001 |
|  |  | Ambient CORT | 0.005 | 0.001 | 3.60 | < 0.001 |
|  | Stress-induced | Intercept | -8.568 | 3.424 | -2.50 | 0.012 |
|  |  | Ambient CORT | 0.004 | 0.002 | 2.41 | 0.016 |

Relationships were not determined (ND) for stress-induced corticosterone of leopard frogs because no larvae had negative stress-induced release rates.

**Supplementary Table 4.** Sample size (*n*), mean, and standard deviation (SD) for baseline and stress-induced waterborne corticosterone (natural log transformed; pg/h) of barred tiger salamanders, northern leopard frogs, and boreal chorus frogs.

|  | **Baseline** | | **Stress-induced** | |
| --- | --- | --- | --- | --- |
| **Species** | ***n*** | **Mean (SD)** | ***n*** | **Mean (SD)** |
| Tiger salamander | 65 | 8.25 (0.46) | 42 | 8.08 (0.40) |
| Leopard frog | 62 | 8.48 (0.81) | 37 | 8.80 (0.86) |
| Chorus frog | 45 | 7.23 (0.51) | 27 | 7.47 (0.48) |

**Supplementary Table 5.** Top-ranked models for baseline and stress-induced waterborne corticosterone (CORT) release rates (pg/h) of tiger salamanders.

| **Measure** | **Num.** | **Model** | ***df*** | **Log lik.** | **AIC_c_** | **ΔAIC_c_** | **AIC_w_** |
| --- | --- | --- | --- | --- | --- | --- | --- |
| Baseline | 1 | Mass | 5 | 16.39 | -21.75 | 0.00 | 0.456 |
|  | 2 | Salinity + Mass + Ambient CORT | 7 | 18.28 | -20.59 | 1.16 | 0.255 |
|  | 3 | Salinity + Mass | 6 | 16.41 | -19.36 | 2.39 | 0.138 |
|  | 4 | Salinity + Mass + Ambient CORT + Salinity × Mass | 8 | 18.49 | -18.41 | 3.34 | 0.086 |
|  | 5 | Salinity + Mass + Salinity × Mass | 7 | 16.93 | -17.89 | 3.86 | 0.066 |
| Stress-induced | 1 | Mass | 5 | 22.36 | -33.06 | 0.00 | 0.574 |
|  | 2 | Mass + Ambient CORT | 6 | 22.59 | -30.78 | 2.28 | 0.184 |
|  | 3 | Salinity + Mass | 6 | 22.38 | -30.36 | 2.69 | 0.149 |
|  | 4 | Salinity + Mass + Ambient CORT | 7 | 22.61 | -27.93 | 5.13 | 0.044 |
|  | 5 | Salinity + Mass + Salinity × Mass | 7 | 22.47 | -27.65 | 5.41 | 0.038 |
|  | 6 | Salinity + Mass + Ambient CORT + Salinity × Mass | 8 | 22.70 | -25.05 | 8.01 | 0.010 |

Models are sorted by corrected Akaike information criterion (AIC_c_) within each measure of CORT with log likelihood (Log lik.), difference in AICc from the best supported model (ΔAIC_c_), and model weights (AIC_w_)

These models (i.e., those with AIC_w_ > 0.001) were included in the top model set and multimodel inference.

**Supplementary Table 6.** Top-ranked models for baseline and stress-induced waterborne corticosterone (CORT) release rates (pg/h) of leopard frogs.

| **Measure** | **Num.** | **Model** | ***df*** | **Log lik.** | **AIC_c_** | **ΔAIC_c_** | **AIC_w_** |
| --- | --- | --- | --- | --- | --- | --- | --- |
| Baseline | 1 | Mass + Ambient CORT | 6 | -30.50 | 74.53 | 0.00 | 0.371 |
|  | 2 | Mass | 5 | -31.85 | 74.76 | 0.23 | 0.330 |
|  | 3 | Salinity + Mass | 6 | -31.58 | 76.69 | 2.16 | 0.126 |
|  | 4 | Salinity + Mass + Ambient CORT | 7 | -30.45 | 76.98 | 2.45 | 0.109 |
|  | 5 | Salinity + Mass + Salinity × Mass | 7 | -31.58 | 79.23 | 4.70 | 0.035 |
|  | 6 | Salinity + Mass + Ambient CORT + Salinity × Mass | 8 | -30.44 | 79.60 | 5.07 | 0.029 |
| Stress-induced | 1 | Salinity + Mass | 6 | -13.19 | 41.17 | 0.00 | 0.736 |
|  | 2 | Salinity + Mass + Salinity × Mass | 7 | -12.91 | 43.68 | 2.50 | 0.211 |
|  | 3 | Mass | 5 | -17.25 | 46.43 | 5.25 | 0.053 |

Models are sorted by corrected Akaike information criterion (AIC_c_) within each measure of CORT with log likelihood (Log lik.), difference in AICc from the best supported model (ΔAIC_c_), and model weights (AIC_w_)

These models (i.e., those with AIC_w_ > 0.001) were included in the top model set and multimodel inference.

**Supplementary Table 7.** Top-ranked models for baseline and stress-induced waterborne corticosterone (CORT) release rates (pg/h) of chorus frogs.

| **Measure** | **Num.** | **Model** | ***df*** | **Log lik.** | **AIC_c_** | **ΔAIC_c_** | **AIC_w_** |
| --- | --- | --- | --- | --- | --- | --- | --- |
| Baseline | 1 | Mass + Ambient CORT | 6 | -11.42 | 37.05 | 0.00 | 0.530 |
|  | 2 | Salinity + Mass + Ambient CORT | 7 | -11.37 | 39.77 | 2.72 | 0.136 |
|  | 3 | Salinity + Mass + Ambient CORT + Salinity × Mass | 8 | -9.90 | 39.80 | 2.75 | 0.134 |
|  | 4 | Mass | 5 | -14.66 | 40.85 | 3.80 | 0.079 |
|  | 5 | Salinity + Mass | 6 | -14.09 | 42.38 | 5.33 | 0.037 |
|  | 6 | Ambient CORT | 5 | -15.48 | 42.49 | 5.44 | 0.035 |
|  | 7 | Salinity + Mass + Salinity × Mass | 7 | -12.82 | 42.66 | 5.61 | 0.032 |
|  | 8 | Salinity + Ambient CORT | 6 | -15.45 | 45.11 | 8.06 | 0.009 |
|  | 9 | (Intercept only) | 4 | -18.62 | 46.25 | 9.20 | 0.005 |
|  | 10 | Salinity | 5 | -18.33 | 48.19 | 11.14 | 0.002 |
| Stress-induced | 1 | Salinity | 5 | -10.00 | 32.86 | 0.00 | 0.496 |
|  | 2 | (Intercept only) | 4 | -12.02 | 33.85 | 1.00 | 0.301 |
|  | 3 | Salinity + Mass | 6 | -9.96 | 36.12 | 3.26 | 0.097 |
|  | 4 | Mass | 5 | -12.02 | 36.89 | 4.03 | 0.066 |
|  | 5 | Salinity + Mass + Salinity × Mass | 7 | -9.01 | 37.92 | 5.06 | 0.039 |

Models are sorted by corrected Akaike information criterion (AIC_c_) within each measure of CORT with log likelihood (Log lik.), difference in AICc from the best supported model (ΔAIC_c_), and model weights (AIC_w_)

These models (i.e., those with AIC_w_ > 0.001) were included in the top model set and multimodel inference.

**Supplementary Table 8.** Summary statistics and model-averaged coefficients (*β* and SE in natural-log scale) for center and scaled predictor variables from linear mixed models for baseline waterborne corticosterone for each amphibian species.

| **Species** | **Variable** | **Num. mod.** | **Importance** | ***β*** | **Adj. SE** | ***z*** | ***p*** |
| --- | --- | --- | --- | --- | --- | --- | --- |
| Tiger | Intercept | 5 | 1.00 | 8.258 | 0.050 | 165.73 | < 0.001 |
| salamander | Mass | 5 | 1.00 | 0.409 | 0.035 | 11.64 | < 0.001 |
|  | Salinity | 4 | 0.80 | -0.002 | 0.045 | 0.06 | 0.957 |
|  | Ambient CORT | 2 | 0.40 | -0.027 | 0.044 | 0.61 | 0.540 |
|  | Mass × Salinity | 2 | 0.40 | -0.007 | 0.028 | 0.24 | 0.808 |
| Leopard | Intercept | 6 | 1.00 | 8.458 | 0.106 | 79.86 | < 0.001 |
| frog | Mass | 6 | 1.00 | 0.666 | 0.057 | 11.67 | < 0.001 |
|  | Salinity | 4 | 0.67 | -0.013 | 0.051 | 0.25 | 0.802 |
|  | Ambient CORT | 3 | 0.50 | 0.072 | 0.095 | 0.76 | 0.449 |
|  | Mass × Salinity | 2 | 0.33 | -0.001 | 0.033 | 0.03 | 0.979 |
| Chorus | Intercept | 10 | 1.00 | 7.222 | 0.118 | 61.21 | < 0.001 |
| frog | Mass | 6 | 0.60 | 0.127 | 0.056 | 2.26 | 0.024 |
|  | Salinity | 6 | 0.60 | 0.026 | 0.159 | 0.17 | 0.869 |
|  | Ambient CORT | 5 | 0.50 | -0.291 | 0.216 | 1.35 | 0.178 |
|  | Mass × Salinity | 2 | 0.20 | 0.012 | 0.033 | 0.37 | 0.714 |

“Num. mod.” is the number of top models that include that predictor variable

“Importance” is the proportions of models that contain that variable

“Adj. SE” is model-adjusted standard error

**Supplementary Table 9.** Summary statistics and model-averaged coefficients (*β* and SE in natural-log scale) for center and scaled predictor variables from linear mixed models for stress-induced waterborne corticosterone for each amphibian species.

| **Species** | **Variable** | **Num. mod.** | **Importance** | ***β*** | **Adj. SE** | ***z*** | ***p*** |
| --- | --- | --- | --- | --- | --- | --- | --- |
| Tiger | Intercept | 6 | 1.00 | 8.119 | 0.071 | 114.78 | < 0.001 |
| salamander | Mass | 6 | 1.00 | 0.389 | 0.034 | 11.51 | < 0.001 |
|  | Salinity | 4 | 0.67 | 0.001 | 0.056 | 0.03 | 0.979 |
|  | Ambient CORT | 3 | 0.50 | -0.012 | 0.059 | 0.20 | 0.840 |
|  | Mass × Salinity | 2 | 0.33 | -0.001 | 0.008 | 0.08 | 0.933 |
| Leopard | Intercept | 3 | 1.00 | 8.799 | 0.076 | 115.96 | < 0.001 |
| frog | Mass | 3 | 1.00 | 0.766 | 0.067 | 11.49 | < 0.001 |
|  | Salinity | 2 | 0.67 | 0.281 | 0.095 | 2.97 | 0.003 |
|  | Mass × Salinity | 1 | 0.33 | -0.022 | 0.081 | 0.27 | 0.789 |
| Chorus | Intercept | 5 | 1.00 | 7.473 | 0.160 | 46.59 | < 0.001 |
| frog | Mass | 3 | 0.60 | 0.005 | 0.038 | 0.14 | 0.891 |
|  | Salinity | 3 | 0.60 | 0.196 | 0.581 | 0.34 | 0.736 |
|  | Mass × Salinity | 1 | 0.20 | -0.004 | 0.023 | 0.16 | 0.874 |

“Num. mod.” is the number of top models that include that predictor variable

“Importance” is the proportions of models that contain that variable

“Adj. SE” is model-adjusted standard error

**Supplementary data II: Tables for Models Omitting Negative Release Rate Observations**

**Supplementary II Table 1.** Top-ranked models for baseline and stress-induced waterborne corticosterone (CORT) release rates (pg/h) of tiger salamanders.

| **Measure** | **Num.** | **Model** | ***df*** | **Log lik.** | **AIC_c_** | **ΔAIC_c_** | **AIC_w_** |
| --- | --- | --- | --- | --- | --- | --- | --- |
| Baseline | 1 | Mass + Ambient CORT | 6 | 14.38 | -14.49 | 0.00 | 0.528 |
|  | 2 | Salinity + Mass | 6 | 13.64 | -13.01 | 1.47 | 0.253 |
|  | 3 | Salinity + Mass + Ambient CORT | 7 | 14.39 | -11.67 | 2.82 | 0.129 |
|  | 4 | Salinity + Mass + Salinity × Mass | 7 | 13.64 | -10.18 | 4.31 | 0.061 |
|  | 5 | Salinity + Mass + Ambient CORT + Salinity × Mass | 8 | 14.39 | -8.67 | 5.81 | 0.029 |
| Stress-induced | 1 | Mass | 5 | 17.43 | -21.52 | 0.00 | 0.697 |
|  | 2 | Salinity + Mass | 6 | 17.63 | -18.33 | 3.19 | 0.141 |
|  | 3 | Mass + Ambient CORT | 6 | 17.48 | -18.01 | 3.51 | 0.121 |
|  | 4 | Salinity + Mass + Salinity × Mass | 7 | 17.72 | -14.44 | 7.08 | 0.020 |
|  | 5 | Salinity + Mass + Ambient CORT | 7 | 17.64 | -14.27 | 7.25 | 0.019 |
|  | 6 | Salinity + Mass + Ambient CORT + Salinity × Mass | 8 | 17.73 | -9.86 | 11.66 | 0.002 |

Models omit observations with negative release rates prior to corrections

Models are sorted by corrected Akaike information criterion (AIC_c_) within each measure of CORT with log likelihood (Log lik.), difference in AICc from the best supported model (ΔAIC_c_), and model weights (AIC_w_)

These models (i.e., those with AIC_w_ > 0.001) were included in the top model set and multimodel inference.

**Supplementary II Table 2.** Top-ranked models for baseline and stress-induced waterborne corticosterone (CORT) release rates (pg/h) of leopard frogs.

| **Measure** | **Num.** | **Model** | ***df*** | **Log lik.** | **AIC_c_** | **ΔAIC_c_** | **AIC_w_** |
| --- | --- | --- | --- | --- | --- | --- | --- |
| Baseline | 1 | Mass | 5 | -23.08 | 57.37 | 0.00 | 0.480 |
|  | 2 | Mass + Ambient CORT | 6 | -22.54 | 58.79 | 1.43 | 0.235 |
|  | 3 | Salinity + Mass | 6 | -23.06 | 59.83 | 2.47 | 0.140 |
|  | 4 | Salinity + Mass + Ambient CORT | 7 | -22.41 | 61.15 | 3.78 | 0.073 |
|  | 5 | Salinity + Mass + Salinity × Mass | 7 | -22.87 | 62.08 | 4.71 | 0.046 |
|  | 6 | Salinity + Mass + Ambient CORT + Salinity × Mass | 8 | -22.06 | 63.18 | 5.82 | 0.026 |
| Stress-induced | 1 | Salinity + Mass | 6 | -12.58 | 40.16 | 0.00 | 0.731 |
|  | 2 | Salinity + Mass + Salinity × Mass | 7 | -12.23 | 42.61 | 2.45 | 0.215 |
|  | 3 | Mass | 5 | -16.64 | 45.35 | 5.19 | 0.055 |

Models omit observations with negative release rates prior to corrections

Models are sorted by corrected Akaike information criterion (AIC_c_) within each measure of CORT with log likelihood (Log lik.), difference in AICc from the best supported model (ΔAIC_c_), and model weights (AIC_w_)

These models (i.e., those with AIC_w_ > 0.001) were included in the top model set and multimodel inference.

**Supplementary II Table 3.** Top-ranked models for baseline and stress-induced waterborne corticosterone (CORT) release rates (pg/h) of chorus frogs.

| **Measure** | **Num.** | **Model** | ***df*** | **Log lik.** | **AIC_c_** | **ΔAIC_c_** | **AIC_w_** |
| --- | --- | --- | --- | --- | --- | --- | --- |
| Baseline | 1 | Salinity + Mass + Ambient CORT | 7 | -2.11 | 22.37 | 0.00 | 0.345 |
|  | 2 | Salinity + Mass + Ambient CORT + Salinity × Mass | 8 | -0.70 | 22.95 | 0.57 | 0.259 |
|  | 3 | Mass | 5 | -5.94 | 23.96 | 1.59 | 0.156 |
|  | 4 | Salinity + Mass + Salinity × Mass | 7 | -3.64 | 25.43 | 3.06 | 0.075 |
|  | 5 | Salinity + Mass | 6 | -5.26 | 25.52 | 3.14 | 0.072 |
|  | 6 | Mass + Ambient CORT | 6 | -5.64 | 26.27 | 3.90 | 0.049 |
|  | 7 | (Intercept only) | 4 | -9.59 | 28.51 | 6.14 | 0.016 |
|  | 8 | Ambient CORT | 5 | -8.48 | 29.02 | 6.65 | 0.012 |
|  | 9 | Salinity + Ambient CORT | 6 | -7.09 | 29.18 | 6.80 | 0.011 |
|  | 10 | Salinity | 5 | -9.52 | 31.12 | 8.74 | 0.004 |
| Stress-induced | 1 | Salinity | 4 | -3.98 | 19.04 | 0.00 | 0.797 |
|  | 2 | Salinity + Mass | 5 | -3.98 | 22.96 | 3.92 | 0.112 |
|  | 3 | (Intercept only) | 3 | -8.37 | 24.45 | 5.42 | 0.053 |
|  | 4 | Salinity + Mass + Salinity × Mass | 6 | -3.07 | 25.77 | 6.73 | 0.028 |
|  | 5 | Mass | 4 | -8.32 | 27.71 | 8.67 | 0.010 |

Models omit observations with negative release rates prior to corrections

Models are sorted by corrected Akaike information criterion (AIC_c_) within each measure of CORT with log likelihood (Log lik.), difference in AICc from the best supported model (ΔAIC_c_), and model weights (AIC_w_)

These models (i.e., those with AIC_w_ > 0.001) were included in the top model set and multimodel inference.

**Supplementary II Table 4.** Summary statistics and model-averaged coefficients (*β* and SE in natural-log scale) for center and scaled predictor variables from linear mixed models for baseline waterborne corticosterone for each amphibian species.

| **Species** | **Variable** | **Num. mod.** | **Importance** | ***β*** | **Adj. SE** | ***z*** | ***p*** |
| --- | --- | --- | --- | --- | --- | --- | --- |
| Tiger | Intercept | 5 | 1.00 | 8.374 | 0.054 | 154.09 | < 0.001 |
| salamander | Mass | 5 | 1.00 | 0.347 | 0.045 | 7.64 | < 0.001 |
|  | Salinity | 4 | 0.80 | 0.007 | 0.046 | 0.15 | 0.878 |
|  | Ambient CORT | 3 | 0.50 | -0.038 | 0.047 | 0.81 | 0.416 |
|  | Mass × Salinity | 2 | 0.40 | -0.001 | 0.022 | 0.02 | 0.981 |
| Leopard | Intercept | 6 | 1.00 | 8.583 | 0.116 | 73.95 | < 0.001 |
| frog | Mass | 6 | 1.00 | 0.622 | 0.058 | 10.74 | < 0.001 |
|  | Salinity | 4 | 0.67 | -0.002 | 0.065 | 0.03 | 0.977 |
|  | Ambient CORT | 3 | 0.50 | 0.033 | 0.072 | 0.46 | 0.644 |
|  | Mass × Salinity | 2 | 0.33 | -0.016 | 0.108 | 0.15 | 0.883 |
| Chorus | Intercept | 10 | 1.00 | 7.433 | 0.060 | 123.18 | < 0.001 |
| frog | Mass | 6 | 0.60 | 0.144 | 0.060 | 2.40 | 0.016 |
|  | Salinity | 6 | 0.60 | 0.124 | 0.320 | 0.39 | 0.698 |
|  | Ambient CORT | 5 | 0.50 | 0.092 | 0.295 | 0.31 | 0.754 |
|  | Mass × Salinity | 2 | 0.20 | 0.026 | 0.047 | 0.56 | 0.579 |

Models omit observations with negative release rates prior to corrections

“Num. mod.” is the number of top models that include that predictor variable

“Importance” is the proportions of models that contain that variable

“Adj. SE” is model-adjusted standard error

**Supplementary II Table 5.** Summary statistics and model-averaged coefficients (*β* and SE in natural-log scale) for center and scaled predictor variables from linear mixed models for stress-induced waterborne corticosterone for each amphibian species.

| **Species** | **Variable** | **Num. mod.** | **Importance** | ***β*** | **Adj. SE** | ***z*** | ***p*** |
| --- | --- | --- | --- | --- | --- | --- | --- |
| Tiger | Intercept | 6 | 1.00 | 8.222 | 0.054 | 151.43 | < 0.001 |
| salamander | Mass | 6 | 1.00 | 0.303 | 0.036 | 8.34 | < 0.001 |
|  | Salinity | 4 | 0.67 | -0.007 | 0.047 | 0.14 | 0.887 |
|  | Ambient CORT | 3 | 0.50 | 0.002 | 0.039 | 0.05 | 0.957 |
|  | Mass × Salinity | 2 | 0.33 | -4.22E-04 | 0.007 | 0.06 | 0.950 |
| Leopard | Intercept | 3 | 1.00 | 8.874 | 0.078 | 113.85 | < 0.001 |
| frog | Mass | 3 | 1.00 | 0.715 | 0.069 | 10.36 | < 0.001 |
|  | Salinity | 2 | 0.67 | 0.286 | 0.096 | 2.97 | 0.003 |
|  | Mass × Salinity | 1 | 0.33 | -0.024 | 0.084 | 0.29 | 0.771 |
| Chorus | Intercept | 5 | 1.00 | 7.681 | 0.083 | 92.06 | < 0.001 |
| frog | Mass | 3 | 0.60 | 0.001 | 0.037 | 0.03 | 0.974 |
|  | Salinity | 3 | 0.60 | 0.231 | 0.102 | 2.27 | 0.023 |
|  | Mass × Salinity | 1 | 0.20 | -0.003 | 0.025 | 0.13 | 0.901 |

Models omit observations with negative release rates prior to corrections

“Num. mod.” is the number of top models that include that predictor variable

“Importance” is the proportions of models that contain that variable

“Adj. SE” is model-adjusted standard error
